# The crosstalk between nerves and immunity: chronic stress as a driver of tumor progression

**DOI:** 10.3389/fimmu.2026.1758894

**Published:** 2026-02-16

**Authors:** Yuan He, Haoliang Yu, Fengze Li, Junzhe Liu, Zhihao Chen, Wenping Zhu, Li Yang, Tengfeng Yan

**Affiliations:** 1 Department of Neurosurgery, The 2nd affiliated hospital, Jiangxi Medical College, Nanchang University, Nanchang, Jiangxi, China; 2 Queen Mary School, University of Nanchang, Jiangxi, Nanchang, China; 3 Jiangxi Key Laboratory of Neurological Tumors and Cerebrovascular Diseases, Nanchang, Jiangxi, China; 4 Jiangxi Health Commission Key Laboratory of Neurological Medicine, Nanchang, Jiangxi, China; 5 Institute of Neuroscience, Nanchang University, Nanchang, Jiangxi, China; 6 The Ministry of Education (MOE) Basic Research and Innovation Center for the Targeted Therapeutics of Solid Tumors, The 2nd affiliated hospital, Jiangxi Medical College, Nanchang University, Nanchang, Jiangxi, China

**Keywords:** cancer, catecholamines, chronic stress, glucocorticoids, hypothalamic-pituitary-adrenal axis, stress management, tumor immunity

## Abstract

Chronic stress, a sustained psychophysiological state, promotes tumor progression primarily by disrupting anti-tumor immunity. Through persistent activation of the HPA axis and sympathetic nervous system, stress hormones such as glucocorticoids and catecholamines reshape the tumor microenvironment and systemically impair immune surveillance. This leads to suppressed activity of cytotoxic lymphocytes, expansion of immunosuppressive cells, and ultimately, enhanced immune evasion and metastasis. Furthermore, these pathways undermine the efficacy of conventional and emerging therapies by fostering multidrug resistance. This review highlights these mechanisms and discusses the promise of targeting stress signaling, through both pharmacological and behavioral interventions, as a strategy to improve cancer outcomes. To address the current lack of clinical guidelines for counteracting the cancer progression mediated by chronic stress, this review propose a tiered screening and intervention model based on easily accessible biostress biomarkers. This hypothesis aims to bridge the gap between basic mechanism research and clinical application, providing a theoretical foundation directional guidance for future research.

## 1 Introduction

Over the past several decades, a profound transformation has been observed in cancer epidemiology, with the disease emerging as the foremost cause of mortality on a global scale (1). Cancer, a significant non-communicable disease posing a substantial public health challenge, predominantly arises from the intricate interplay between environmental determinants and genetic predispositions (2). Advanced genomic sequencing initiatives have unveiled the complex genomic landscape of human cancers, highlighting that specific intragenic mutations, known as “driver mutations,” can instigate or “drive” tumorigenesis (3). A typical tumor harbors 2 to 8 such “driver gene” mutations, which confer a selective growth advantage, while the remaining mutations are considered “passenger” mutations, making no significant contribution to tumor progression. Epidemiological investigations have pinpointed a multitude of environmental carcinogens, spanning from infectious agents to modifiable lifestyle factors such as tobacco use, alcohol consumption, obesity, and unhealthy dietary and physical activity patterns (4). Notably, beyond established risks such as smoking and diet, chronic psychological stress is increasingly recognized as a key regulator of cancer progression, primarily through its disruption of antitumor immunity.

Stress encompasses a spectrum of neuroendocrine responses, predominantly characterized by sympathetic nervous system excitation and augmented pituitary-adrenocortical secretion (5). It also encompasses a suite of nonspecific reactions that the body initiates when confronted with intense stimuli (6). Recent narrative review has further provided a detailed description of this perspective, highlighting the mechanisms whereby stress-mediated neuro-endocrine pathways result in allostatic overload, thereby manifesting the substantial research significance in this domain (7). Stress is categorized into acute and chronic forms. Acute stress typically arises during emergencies, such as when faced with threats or challenges, and it manifests as an enhancement of protective mechanisms. This transient response is generally advantageous for the body (8). In contrast, chronic stress results from an individual’s prolonged and repeated exposure to various stressors over an extended period. The physiological ramifications of chronic stress include but are not limited to, the sustained activation of the neuroendocrine system (9). Specifically, it involves the hyperactivation of the hypothalamic-pituitary-adrenal (HPA) axis, leading to a chronically elevated release of cortisol and other stress hormones (10). This neuroendocrine cascade directly orchestrates immunosuppression by dysregulating immune cell function and disrupting the cytokine network, thereby fostering a permissive microenvironment for tumor development (11).

Chronic stress has a profound effect on the overall risk of developing cancer, which is particularly pronounced in men and shows a worrying trend towards younger age groups (12, 13). For example, chronic stress, especially social stress, significantly increases the risk of breast cancer and exacerbates the disease phenotype (14). With the research in this field continues to advance, chronic stress has increasingly been demonstrated to exert pro-tumorigenic effects across multiple cancer types, through mechanisms such as enhanced tumor growth, metastasis, and therapy resistance (15). Epidemiologic studies have shown that cancer patients are more likely to suffer from depression and anxiety compared to the general population, which is complementary with the idea that chronic stress contributes to cancer incidence and progression (16, 17). In recent years, scientific studies have increasingly focused on the role of chronic stress in cancer development. A large body of evidence suggests that chronic stress is not only a potential risk factor for cancer development but may also promote tumor growth, invasion and metastasis (18). This review aims to synthesize the mechanisms by which chronic stress subverts anti-tumor immunity to drive cancer progression. We will focus on how stress hormones remodel the tumor immune microenvironment, inhibit systemic immune surveillance, and ultimately compromise therapeutic efficacy, thereby highlighting potential avenues for intervention.

## 2 Materials and methods

A search of the related literature was conducted on the PubMed database using the keywords “Neuroimmune Interaction”, “Chronic Stress”, and “Tumor Progression”, which yielded the following search criteria:((“neuroimmune interaction”[All Fields] OR “neural immunity”[All Fields] OR “neuroimmunity”[All Fields] OR “Neuroimmune Interactions”[MeSH Terms] OR “nerve immunity crosstalk”[All Fields]) AND (“chronic stress”[All Fields] OR “chronic psychological stress”[MeSH Terms] OR “chronic stress disorder”[All Fields]) AND (“tumor progression”[All Fields] OR “neoplasm progression”[MeSH Terms] OR “cancer progression”[All Fields] OR “metastasis”[All Fields]) AND 2010/01/01:2025/12/31[Date-Publication]) AND (english[Filter]).

## 3 Results

**Figure 1:**
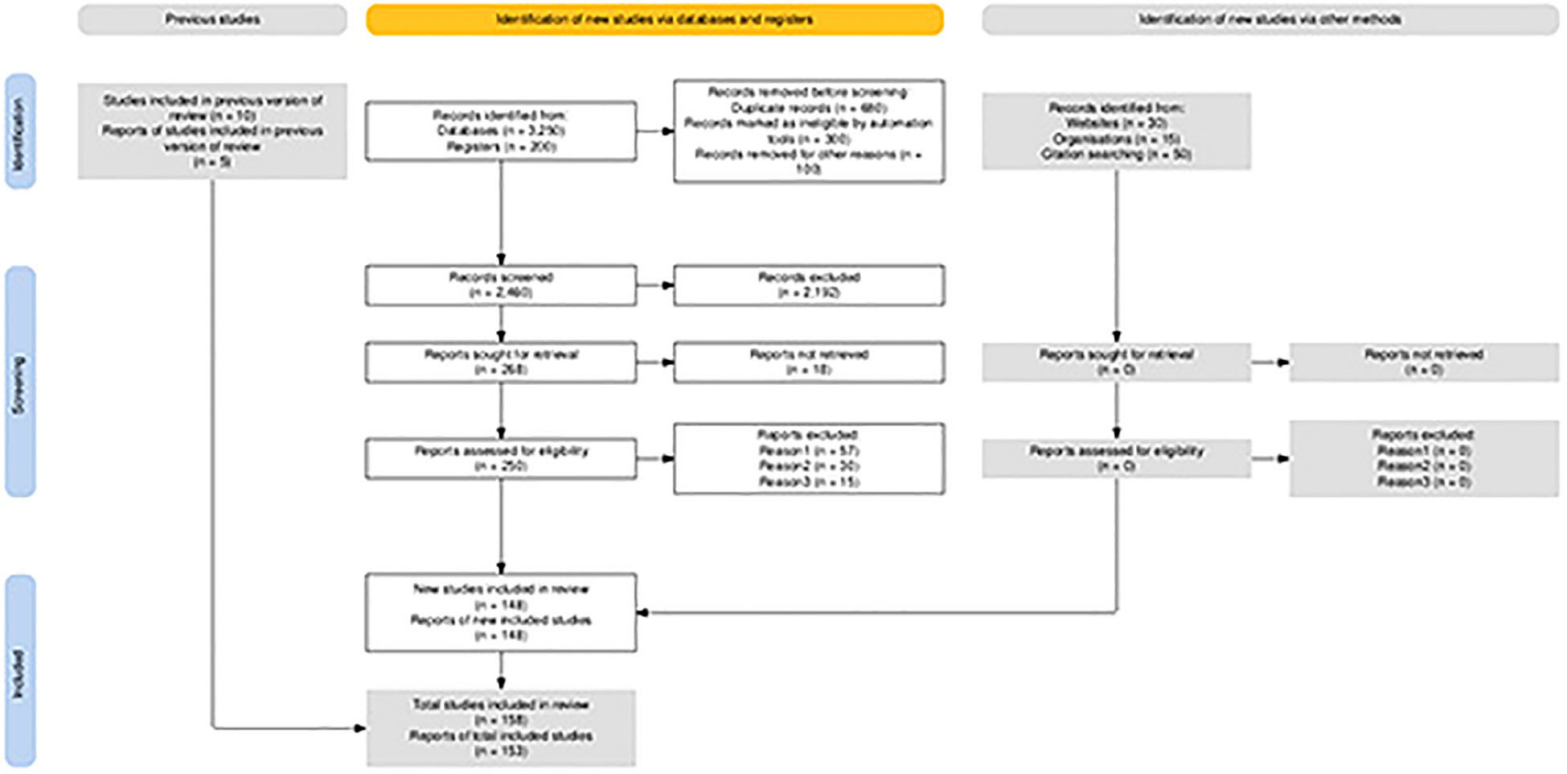
Prisma table showing the search criteria and selection process for this review. It provides transparency on the systematic approach used in literature screening and inclusion, though this review does not fully adhere to all the requirements of a systematic review.

Literature not directly related to the impact of chronic stress on tumor progression was excluded. The authors acknowledged that this retrieval strategy might have inadvertently missed important research papers. **Figure 1** presents the PRISMA table, which further illustrates the research sampling procedures by systematically listing the number of literature and key bases at each stage of retrieval, deduplication, screening, and final inclusion, thereby enhancing the transparency and traceability of the literature screening process (19).

## 4 Physiopathological mechanisms of chronic stress

### 4.1 Chronic stress promotes the secretion of related hormones

**Figure 2:**
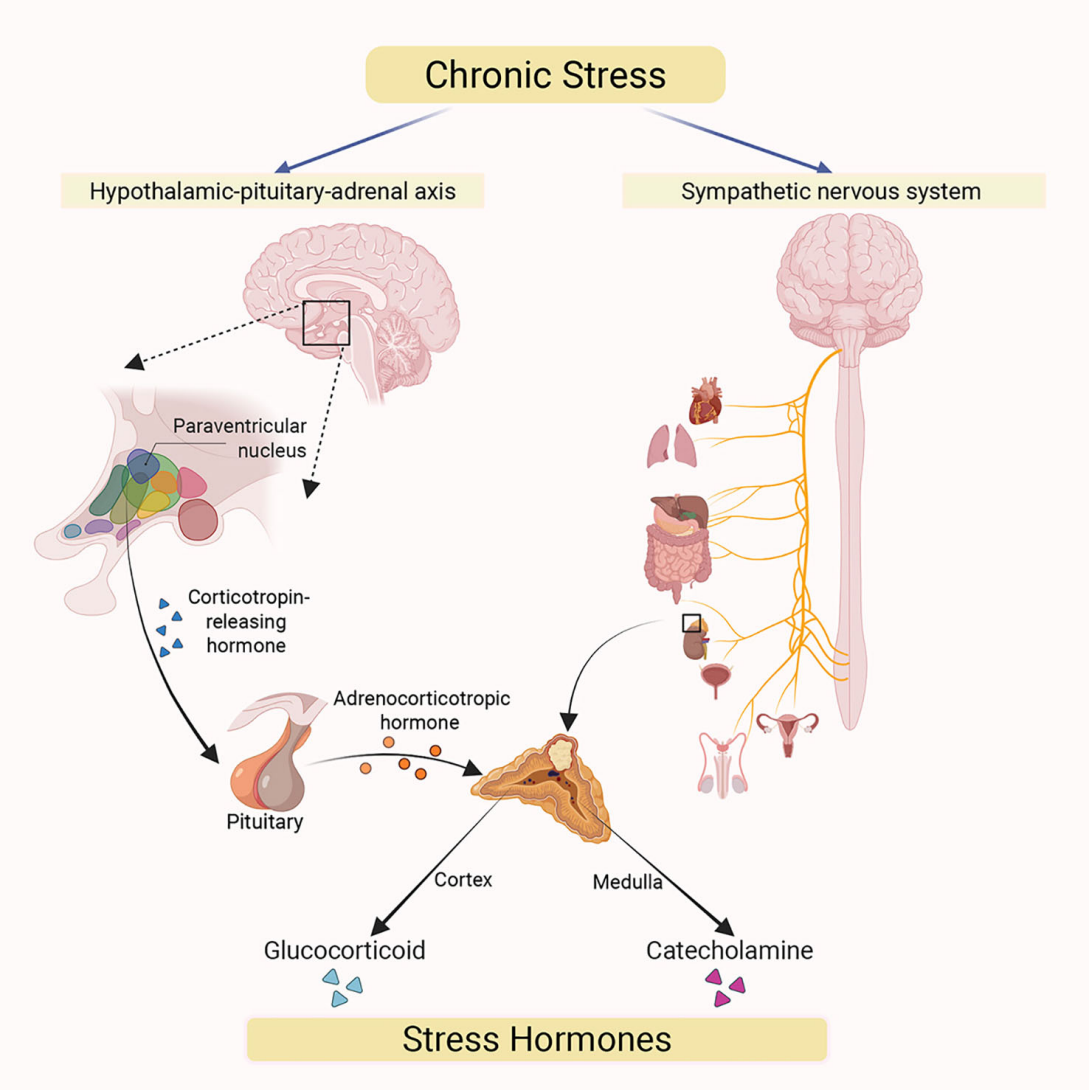
Chronic stress acts on two major efferent pathways originating in the brain. The hypothalamic-pituitary-adrenal (HPA) axis (left): Chronic stress triggers the release of corticotropin-releasing hormone (CRH) from paraventricular nucleus (PVN) of the hypothalamus, which acts on the pituitary gland. The pituitary then secretes adrenocorticotropic hormone (ACTH) into systemic circulation, stimulating the synthesis and secretion of glucocorticoids from adrenal cortex. The sympathetic nervous system (SNS) (right): Chronic stress activates the SNS, whose nerve fibers directly innervate the adrenal medulla, promoting the secretion of catecholamine hormones (primarily epinephrine and norepinephrine). Under chronic stress, both glucocorticoids and catecholamines are released into the systemic circulation through these two pathways. This results in elevatedcirculating stress hormone levels, which subsequently mediate a range of systemic physiological responses.

Chronic stress has been extensively documented to induce the production of stress hormones, such as catecholamines and glucocorticoids (20). This induction primarily proceeds via two distinct pathways (**Figure 2**). First, the activation of the hypothalamic-pituitary-adrenal (HPA) axis stimulates the release of glucocorticoids. Specifically, chronic stress activates the paraventricular nucleus (PVN) of the hypothalamus to secrete corticotropin-releasing hormone (CRH) (21). Subsequently, it binds to specific receptor, corticotropin-releasing hormone receptor 1 (CRHR1), on the corticotroph cells of the anterior pituitary which triggers the synthesis and release of adrenocorticotropic hormone (ACTH). Furthermore, ACTH binds to receptors on cells located in the zona fasciculata of the adrenal cortex, activating the biosynthesis and secretion of glucocorticoids. Meanwhile, chronic stress can also activate the sympathetic nervous system (SNS), which in turn stimulates chromaffin cells (CCs) in the adrenal medulla to release catecholamine hormones such as epinephrine (E) and norepinephrine (NE) (20). These CCs contain abundant secretory chromaffin granules (CGs), which store catecholamines and release them upon SNS activation (22). The catecholamines released by CCs subsequently enter the systemic circulation to exert their physiological effects (23).

### 4.2 Immunomodulatory effects of stress hormones

**Figure 3:**
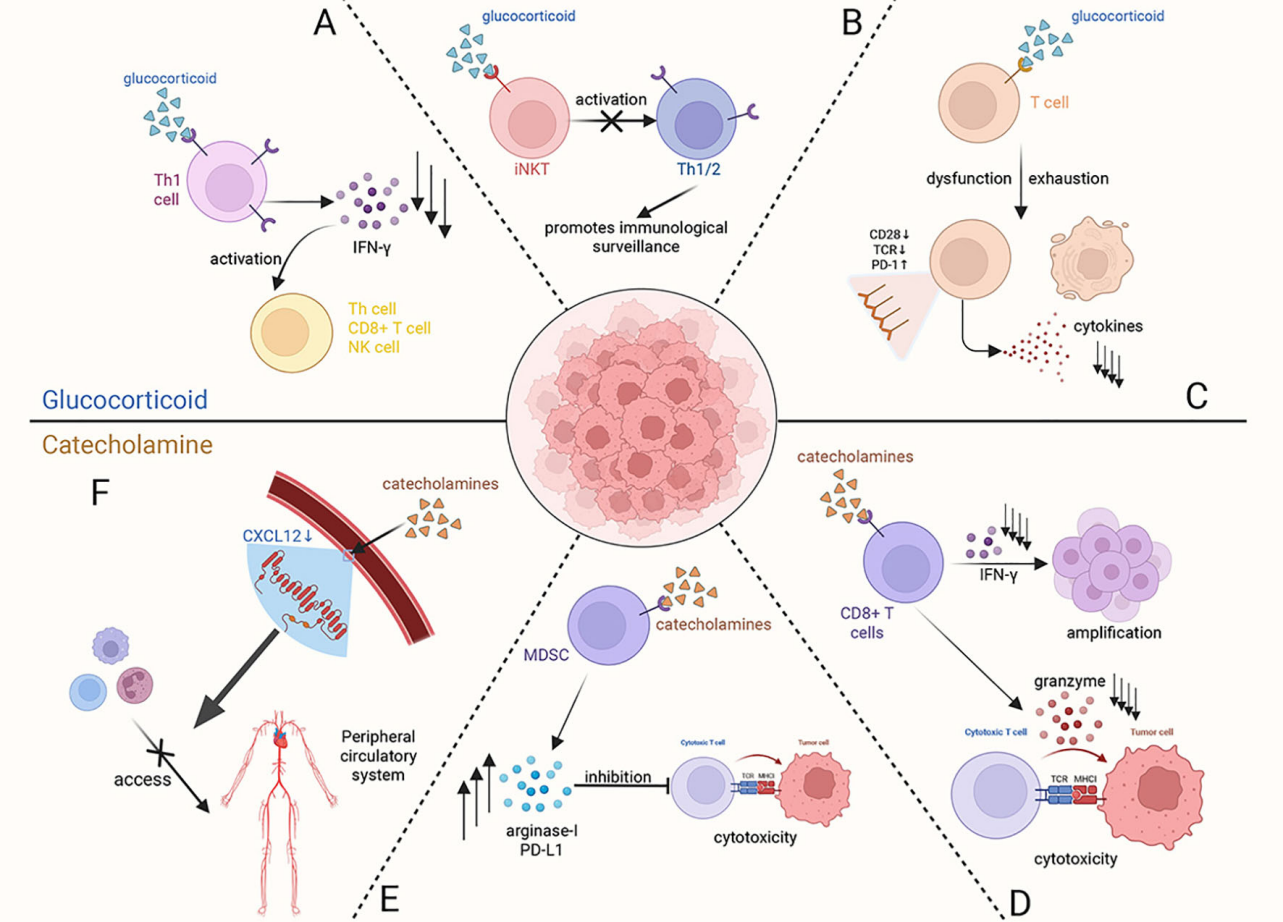
At the systemic level, glucocorticoids and catecholamines, as key stress hormones, exert a synergistic suppressive effect on the immune system by acting on various immune cells. **(A)** Glucocorticoids act on Type 1 helper T cells (Th1), downregulating the secretion of IFN-γ to inhibit the activation of Th cells, CD8+ T cells, and natural killer (NK) cells. **(B)** Glucocorticoid receptor (GR) signaling induces dysfunction of invariant natural killer T (iNKT) cells, indirectly impairing the normal reactivity of Th1/Th2 cells, which ultimately compromises immune surveillance. **(C)** Activation of the GR signaling pathway within T cells leads to their dysfunction and exhaustion, altering the expression of immune-related molecules on the cell surface and even inducing T cell apoptosis. **(D)** Catecholamines act on CD8+ T cells, inhibiting their proliferation by reducing IFN-γ secretion and decreasing cytotoxicity by suppressing the release of granzymes. **(E)** Myeloid-derived suppressor cells (MDSCs), when acted upon by catecholamines, promote the synthesis and secretion of immunosuppressive molecules (such as Arginase-I and PD-L1), which in turn act on cytotoxic T cells to suppress their cytotoxic function. **(F)** Elevated levels of catecholamines induce a decreasing of CXCL12 expression in peripheral vascular endothelial cells, thereby blocking the migration of immune cells toward peripheral circulatory system.

Stress hormones, including glucocorticoids and catecholamines, can exert detrimental effects on the systemic immune system under conditions of chronic stress. These immunosuppressive responses may ultimately enable cancer cells to evade immune surveillance, thereby promoting carcinogenesis and metastasis (**Figure 3**).

Current research indicates that glucocorticoids negatively impact the immune system by binding to glucocorticoid receptors (GRs) on immune cells, thereby disrupting their normal biological functions. Furthermore, it reduces the production of interferon-gamma (IFN-γ), a critical cytokine that activates cytotoxic immune responses by enhancing the activation of type 1 helper T cells, CD8+ T cells, and natural killer cells (24). GR signaling also contributes to the dysregulation of invariant natural killer T (iNKT) cells, thereby suppressing both Th1- and Th2-type immune responses, which weakens cytotoxic immunity against tumors (25). Except directly impairing cytotoxic immune responses, excessive glucocorticoid secretion has also been implicated in promoting T cell exhaustion, which is associated with poor prognosis (26). As for catecholamines, they can also directly affect CD8^+^ T cells via β1-adrenergic receptors (β1-ARs), suppressing the secretion of IFN-γ and granzyme, which both are essential for T cell proliferation and cytotoxic function (27). In stance, this specific binding induces the dysfunction and exhaustion of CD8+ tumor-infiltrating lymphocytes (TILs), which significantly impairs anti-tumor immunity (28). Meanwhile, catecholamines can also act on β2-adrenergic receptors (β2-ARs) found on myeloid-derived suppressor cells (MDSCs). This interaction alters their synthesis and secretion of immunosuppressive molecules like arginase-I and PD-L1 through STAT3 signal pathway, ultimately suppressing the anti-tumor function of T cells (29, 30). As a key catecholamine, high levels of NE released from sympathetic nervous system (SNS) nerve endings in the bone marrow microenvironment can also inhibit the expression of CXCL12 to sequester neutrophils and monocytes away from the peripheral blood system, consequently suppressing the immune response (31). Generally, these mechanisms demonstrate that stress-induced catecholamines contribute to inhibit the immune system surveillance against tumor cells.

## 5 Epidemiological evidence

A large number of epidemiological studies have revealed significant associations between chronic stress states, such as depression and anxiety, and a poor prognosis for tumors (**Table 1**). A systematic review showed that the prevalence of depression in cancer patients was as high as 12.5%, which is four times higher than in the general population (32). Similar trends have been found in epidemiological studies of anxiety. Notably, anxiety symptoms had a particularly significant and consistent effect on gender-related cancers. A multicenter cohort study showed that patients with bladder and testicular cancer exhibited higher levels of anxiety. Compared to the general population, bladder cancer patients were 5.3 times more likely to experience anxiety, while testicular cancer patients were 5.0 times (33). Furthermore, several studies have demonstrated a higher prevalence of anxiety symptoms among breast cancer patients, with odds ratios (OR) ranging from 2 to 6. In addition to its effects on tumor formation, a follow-up study at the MD Anderson Cancer Center found that patients with a chronic negative mood had a 32% higher rate of cancer recurrence and significantly higher levels of inflammatory factors in their serum (e.g. IL-6) than the control group (11, 34). Elevated IL-6 levels were notably strongly associated with the progression of eight malignant tumors, including breast and colorectal cancer. An epidemiological survey of 8,387 participants aged 51–61 found that those diagnosed with cancer were at the highest risk of developing depressive symptoms within two years of their initial diagnosis compared to individuals diagnosed with common chronic diseases such as diabetes, hypertension, and heart disease (35). This suggests that chronic stress may affect tumor progression to some extent. However, rigorously designed prospective cohort studies are still needed to validate this association further.

**Table 1:** The association between chronic stress and cancer risk.

Chronic stress type	Gender	Risk change	Cancer type	Ref.
Depression	Both	↑15%	All	(36)
Depression	Both	↑20%	Liver cancer	(36)
Depression	Both	↑33%	Lung cancer	(36)
Depression	Female	↑23%	Breast cancer	(45)
Anxiety	Both	↑29%	All	(32)
Anxiety	Both	↑113%	Thyroid cancer	(32)
Anxiety	Male	↑97%	Prostate cancer	(32)
Anxiety	Female	↑19%	Breast cancer	(45)
Work stress	Both	↑36%	Colorectal cancer	(37)
Work stress	Both	↑24%	Lung cancer	(37)
Work stress	Both	↑112%	Esophageal cancer	(37)
Work stress	Male	↑40%	Prostate cancer	(49)
Childhood trauma	Both	↑90%	Brain tumor	(38)
Bereavement	Female	↑31%	Breast cancer	(62)
Major stressful life events	Male	↑221%	Lung cancer	(51)
Psychosocial stress	Female	↑250%	Cervical cancer	(39)
Cumulative stress	Female	↑509%	Breast cancer	(44)
Occupational frustration	Male	↑61%	Prostate cancer	(48)
Marital separation	Male	↑94%	Prostate cancer	(48)
Being highly sensitive to others’ evaluations	Male	↑73%	Prostate cancer	(48)

Several meta-analyses and large cohort studies have confirmed that chronic stress has a significant, heterogeneous effect on tumor progression. This heterogeneity manifests in two main aspects: firstly, different forms of chronic stress have entirely different effects on tumors, and secondly, the same type of stress affects different tumors differentially. Specifically, the pooled results of 24 studies revealed that depressive symptoms were associated with a 15% increase in overall cancer risk, a 20% increase in liver cancer risk, and a 33% increase in lung cancer risk (36). Job stress was also found to significantly increase the risk of colorectal, lung and esophageal cancers (37). While anxiety and depression are common symptoms of chronic stress, there are other types of chronic stress. For instance, childhood trauma can increase the risk of brain tumors by 90% (38). Furthermore, studies of cervical precancerous lesions have found that psychosocial stress, such as marital conflict, increases lesion risk by 2.5-fold and that this effect is independent of traditional risk factors, such as human papillomavirus (HPV) infection (39). These results were validated in twin cohort studies: major life events such as divorce or bereavement increased women’s breast cancer risk by 40% (40). Furthermore, when occupational chronic psychological stress levels were high, serum heat shock protein 70 (HSP70) expression levels decreased while tumor necrosis factor-α (TNF-α) expression levels increased, suggesting a decrease in the organism’s emergency response capacity (41).

There are prominent differences between men and women in terms of how they perceive stress and their biological responses to it. These differences can impact cancer incidence, development and heterogeneity (42). The link between chronic stress and female tumors, particularly breast and gynecological tumors, shows a significant cumulative effect. Depressive symptoms can triple the risk of treatment non-adherence in breast cancer patients, and preoperative depressive symptoms are strongly associated with significantly elevated IL-6 levels following surgery (43). Studies have also found that major life events, such as the death of a loved one, personal injury or illness, and legal disputes, can significantly increase the risk of breast cancer. Individuals with a cumulative stress score of over 210 have a 5.09-fold increased risk of breast cancer (44). Furthermore, a prospective cohort study revealed that women who experienced the death of their mother before the age of 20 exhibited a 31% increased risk of breast cancer (45). In breast cancer patients, increased tumor-infiltrating B cells and T cells were significantly associated with criminal exposure, anxiety symptoms and exposure to adverse childhood events (46). In ovarian cancer patients, social isolation is associated with higher levels of norepinephrine in the tumor microenvironment, which may promote malignant tumor progression, indicating that depression, anxiety and socio-environmental adversity may be potential modifiers of the tumor immune microenvironment for women patients (47). Chronic stress and tumors have distinct patterns of association in men. A related case-control study found that occupational frustration, marital separation and being highly sensitive to others’ evaluations significantly increased the risk of prostate cancer (48). Another study showed that men with job stress lasting more than 30 years had a 40% increased risk of prostate cancer (49). Throughout treatment, men with prostate cancer exhibited a relatively high prevalence of depression and anxiety. Interestingly, there was a rebound increase in the prevalence of depression and anxiety after treatment compared to before, possibly due to anxiety specific to postoperative prostate cancer, such as psychological distress resulting from decreased sexual function (50). Meanwhile, men who had experienced a major stressful event in the previous five years were 2.21 times more likely to develop lung cancer (51).

However, epidemiological findings should be interpreted with caution. Reverse causality and behavioral confounders may exaggerate the strength of the association between psychological stress and cancer (52). For example, chronic stress may promote gastric cancer invasion and metastasis by activating the SNS and HPA axis, and is therefore a potential pathogenic factor. Meanwhile, gastric cancer itself and its treatment (e.g. gastrectomy) can also induce depressive symptoms (53). Studies have shown that approximately 57% of gastric cancer patients experience depression, creating a vicious cycle of “stress-cancer-stress” (54). Notably, various confounding factors, including behavioral factors and differences in access to healthcare resources, often confuse such studies. For instance, some studies have demonstrated that, while cancer risk is elevated in populations experiencing depression, the strength of the association between depression and cancer is significantly reduced when behavioral factors such as smoking and alcohol abuse are taken into account (44). Therefore, behavioral factors must be adequately considered in future multivariate statistical models.

## 6 The relationship between chronic stress and cancer progression

**Figure 4:**
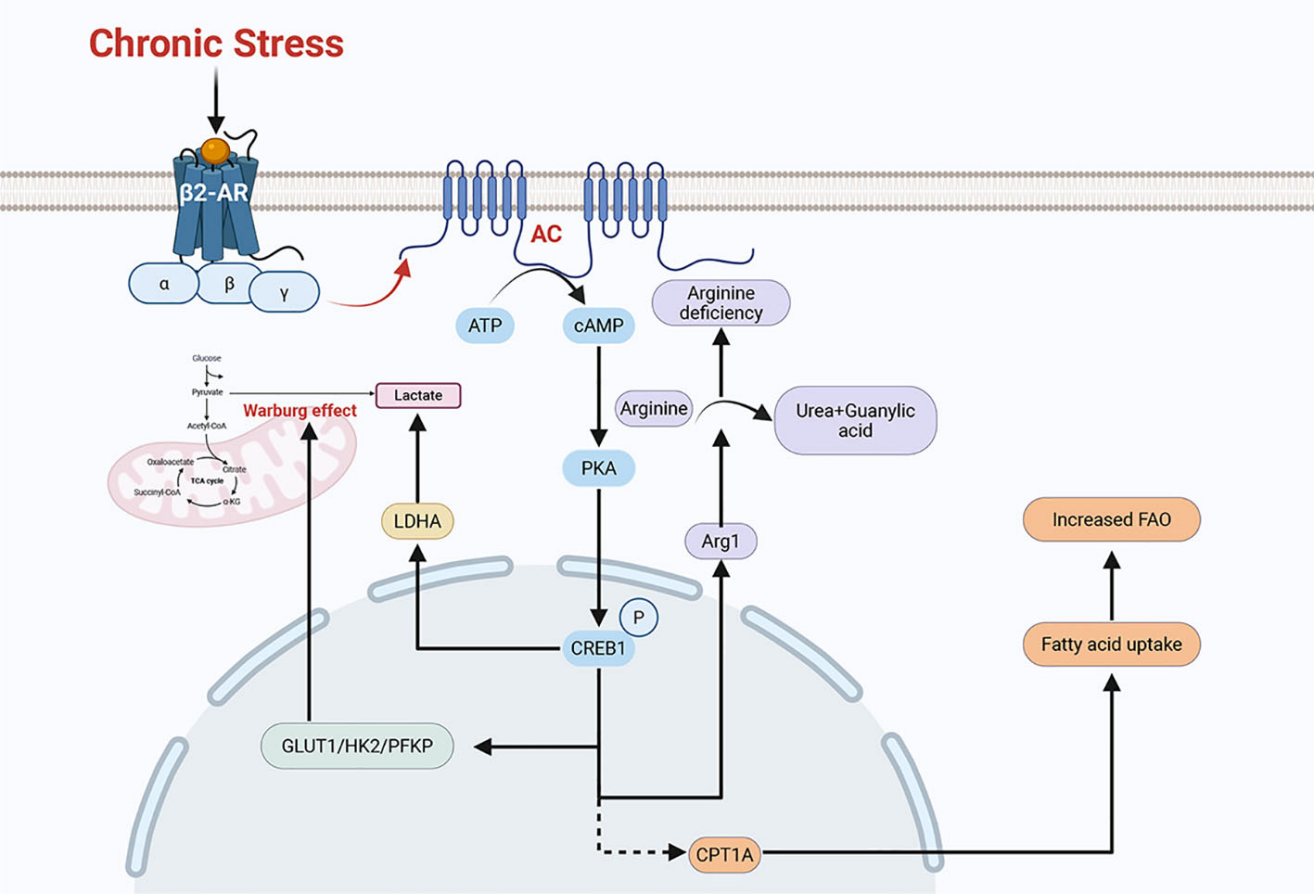
Chronic stress can remodel the regulatory pathways of multiple metabolic processes. Chronic stress induces the phosphorylation of CREB1 via the β2-AR/PKA/CREB1 pathway, thereby upregulating the expression of key glycolytic enzymes such as GLUT1, HK2, and PFKP. This promotes aerobic glycolysis (the Warburg effect) in tumor cells, leading to lactate production. Additionally, chronic stress can induce the activation of LDHA (lactate dehydrogenase) by adrenaline and ultimately generate lactate. Chronic stress can activate the β-AR signaling pathway to polarizes macrophages toward an M2-like phenotype. These M2-polarized TAMs (tumor-associated macrophages) express arginase 1 (Arg1), which hydrolyses arginine into ornithine and urea, creating an arginine-deficient microenvironment. The chronic stress-activated β2-AR signaling pathway also upregulates the expression of the fatty acid transporter CPT1A. This upregulation of CPT1A promotes fatty acid uptake and FAO (fatty acid oxidation). These remodeled metabolic pathways can either promote tumor growth or induce immunosuppression, ultimately contributing to tumor progression.

**Figure 5:**
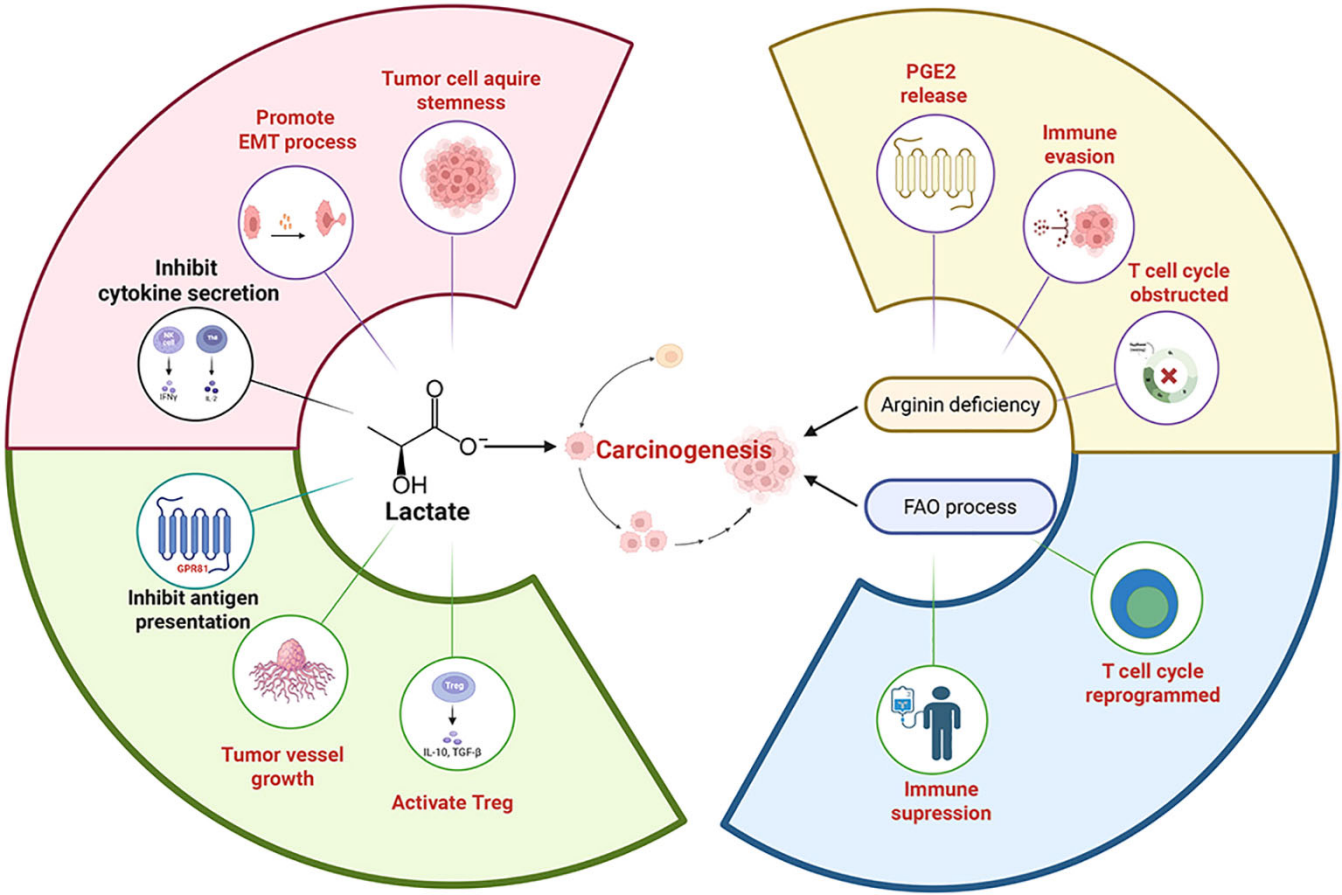
The metabolic pathways remodeled by chronic stress can induce numerous physiological changes. Lactate, a product of chronic stress-remodeled metabolic pathway, can activate the GPR81 protein on immune cells, which in turn prevents the presentation of tumor-specific antigens to other immune cells, promotes angiogenesis and facilitates immune evasion. Lactate can also be taken up by Tregs (regulatory T cells), enhancing their immunosuppressive function and thereby promoting tumor growth. The pH decrease caused by lactate can also promote tumor development. For example, a low pH canfacilitate the development of cancer stem-like properties through SLUG, and can also induce EMT (epithelial-mesenchymal transition), which increases the migratory capacity of tumor cells. The acidified microenvironment can also suppress the production of cytokines (e.g., IFN-γ, IL-2) and inhibit cytotoxic function. In the arginine-deficient microenvironment induced by chronic stress, activated T cells undergo metabolic and transcriptional reprogramming via the ATF4-SLC7A11-GSH axis, which suppresses their immune function. In the absence of L-arginine, T cells become arrested in the G0-G1 phase of the cell cycle and are unable to proliferate continuously. These changes lead to immunosuppression and promote tumor progression. Tumor cell CPT1Amediated fatty acid oxidation (FAO) acts as an “anti-elimination switch.” Sufficient FAO allows tumor cells to maintain survival signals under the “cytolytic pressure” exerted by immune cells, thus promoting tumor cell immune evasion. Furthermore, FAO in tumor cells can increase PGE2 release, which suppresses immune responses and facilitates tumor development.

Experiments indicate that under chronic stress conditions, excessive secretion of stress hormones can promote the proliferation, migration, and metastasis of tumor cells (52). These promotion effects are mainly mediated by modulating the tumor microenvironment (55). Chronic stress can reprogram metabolic pathways and modify the composition and properties of the tumor microenvironment (**Figure 4**). These changes contribute to immunosuppression and immune evasion, thereby promoting tumor progression (**Figure 5**). Collectively, the mechanisms described above constitute a set of complex and parallel pathways through which chronic stress exerts its pro-tumorigenic effects (**Table 2**).

**Table 2:** Key molecular mediators and mechanisms by which chronic stress triggers tumor progression and therapy resistance.

Stress factor	Target sensor	Target cell	Mechanism	Consequence	Ref
*Catecholamine*	β-AR	Tumor Cells	Activates STAT1/IRF1 axis.	Impairs antigen processing.	(89)
	β-AR	Tumor Cells	Upregulates TRIM2.	Inhibits apoptosis.	(124)
	β2-AR	Tumor Cells	Phosphorylates CREB1.	Enhances aerobic glycolysis.	(56)
	β2-AR	Tumor Cells	Activates the Arachidonic Acid cycle.	Suppresses immunity.	(30, 77)
	β2-AR	Tumor Cells	Increases LDHA expression.	Impairs immune cell function.	(116)
	β2-AR	Tumor Cells	Upregulates transcription factor Cx32.	Promotes resistance to EGFR-TKIs.	(114, 115)
	β2-AR	Macrophages (TAMs)	Macrophage Polarization: Shifts M1 to M2 phenotype.	Suppresses T cells.	(67, 68)
	β2-AR	MDSCs	Regulates the synthesis of immunosuppressive molecules.	Suppresses cytotoxic T-cell function.	(29, 30)
	β1-AR	CD8+ T Cells	Inhibits IFN-γ and granzyme secretion; Activates MAPK to upregulate PD-L1.	Inhibits TCR signaling.	(27, 89)
	β1-AR	Tumor Cells/T Cells	Phosphorylates eIF2α and activates p38 MAPK.	Leads to elevated PD-L1 levels.	(89)
	cAMP/PKA	M2 TAMs/DCs	Upregulates CCL18 secretion.	Induces differentiation and recruitment of Tregs.	(103, 104)
	N/A	Bone Marrow Nerves	Inhibits CXCL12 expression in bone marrow niches.	Sequesters neutrophils/monocytes in bone marrow.	(31)
*Glucocorticoid*	GR	iNKT Cells	Binds intracellular GR to disrupt cytokine balance.	Suppresses cytotoxic immunity.	(25)
	GR	DCs	Induces Tsc22d3 expression.	Impairs T-cell activation.	(101)
	GR	Microglia/Neutrophil	Stimulates HMGB1 release.	Promote metastasis.	(105–107)
*Lactate*	GPR81	Tumor Cells/DCs	Activates GPR81.	Facilitates immune evasion.	(58–60)
	N/A	Tumor Cells	Enhances HIF-1α stability.	Promotes angiogenesis.	(61, 62)
	MCT1	Tregs	Tregs take up lactate via MCT1.	Enhances Treg immunosuppressive function.	(63)
*Acidification*	N/A	T Cells/NK Cells	Prevents NFAT upregulation;.	Prevents lysis of tumor cells	(64, 66)
	N/A	Tumor Cells	Upregulates BCRP.	Reduces drug accumulation.	(119, 120)
*Amino Acid Depletion*	Low Arginine	T Cells	Downregulates CD3ζ chain.	Impairs T cell proliferation and cytokine secretion.	(158)
	ATF4	CD4+ T Cells	Activated under arginine deficiency conditions.	Induces Treg-like immunosuppressive properties.	(70)
	AhR	Tregs	Activates Aryl Hydrocarbon Receptor.	Promotes tolerance.	(71, 73)
*ROS*	N/A	T Cells	Causes excessive mitochondrial ROS accumulation.	Inhibits T-cell proliferation and induces exhaustion.	(85–87)
*Immune Checkpoint Receptors*	TIM-3	T Cells	Upregulates TIM-3 and its ligand Galectin-9.	Promotes T cell autophagy and apoptosis.	(92)
	LAG-3	CD8+ T Cells	Upregulates LAG-3.	Causes metabolic dysregulation.	(94)
*CD36*	N/A	Tregs/Tumor Cells	Promotes ECM adhesion.	Induces EMT and vascular mimicry.	(80, 82)
	N/A	MDSCs	Sustains FAO specifically in MDSCs.	Preserves the immunosuppressive function.	(82)

### 6.1 Disruption of glucose metabolism and lactate homeostasis, leading to immunosuppression, immune evasion and tumor progression

Chronic stress can influence the glucose metabolism of tumor cells. For example, chronic stress reprograms metabolic pathways and enhances glycolysis (the Warburg effect) in a variety kinds of tumor cells (56). Besides, chronic stress can also increase the expression of glycolysis-related enzymes, including GLUT1, HK2, and PFKP. Chronic stress can activate the β2-AR/PKA/CREB1 pathway, which phosphorylates CREB1 to increase the expression of glycolytic enzymes (56). Glycolysis (the Warburg effect) in tumor cells produces lactate. Chronic stress-induced adrenaline activates lactate dehydrogenase(LDHA), which can also produce lactate and ultimately leads to a decrease in pH (57). This has a significant effect on tumor immune microenvironment.

#### 6.1.1 Lactate-mediated signaling via GPR81

Lactate is a regulator of the immune system and can mediate a series of immune-evasive and immunosuppressive responses. Lactate can act as an agonist for the G protein-coupled receptor GPR81, acting as a signaling molecule, the signaling pathways involve autocrine and paracrine mechanisms (58, 59). In the autocrine pathway, lactate produced by cancer cells activates GPR81 on the cancer cells, inducing the production of PD-L1 in tumor cells, thereby tumor cells can evade the immune system (60). In the paracrine pathway, lactate produced by cancer cells activates GPR81 on immune cells (such as dendritic cells) present in the tumor stroma, which inhibits the cell surface presentation of MHC II. The activation of GPR81 in dendritic cells can prevent tumor-specific antigens from being presented to other immune cells, ultimately leading to angiogenesis and immune evasion (58–60). Lactate can also enhance HIF-1α stability, promote VEGF expression and tumor angiogenesis to promote tumor progression (61, 62).

#### 6.1.2 Metabolic support for Tregs and T cell suppression

Lactate can also directly affect the function of immune cells, thereby promoting immunosuppression and immune evasion of tumor cells. Tregs (regulatory T cells) efficiently take up lactate through MCT1, supporting their TCA cycle and gluconeogenesis, thereby enhancing the immunosuppressive function of regulatory T cells (63). Lactate itself is also an effective inhibitor of cytotoxic T cell and NK cell function and survival. High concentrations of lactate prevent the upregulation of nuclear factor of activated T cells (NFAT) in cytotoxic T cells and NK cells, leading to reduced IFN-γ production (64). The reduction in IFN-γ secretion weakens the killing function of immune cells (65). Lactate can also promote the expression of NKG2D ligands on monocytes, which leads to the downregulation of NKG2D on natural killer (NK) cells, preventing tumor-infiltrating NK cells from lysing tumor cells that express NKG2D ligands and thereby promoting immune evasion (66). The acidification of the microenvironment (pH below the normal range) caused by lactate secretion disrupts the normal metabolism of CD8^+^ T cells and NK cells, inhibiting glycolysis, proliferation, cytokine production (such as IFN-γ, IL-2), and cytotoxic function of CD8+ T cells (63, 65). In summary, lactate itself and the resulting acidification of the tumor microenvironment exert a significant impact on the tumor immune microenvironment, leading to immunosuppression and immune escape, ultimately promoting tumor progression.

### 6.2 Chronic stress disrupts amino acid metabolism, leading to immunosuppression and tumor progression

#### 6.2.1 Macrophage polarization and arginine depletion

Chronic stress activates β-adrenergic signaling, disturbing the polarization balance of macrophages within tumor tissues, shifting them from the M1 toward the M2 phenotype, thereby increasing the population of M2 tumor-associated macrophages (TAMs) (67, 68). M2 TAMs express arginase-1 (Arg1), which hydrolyzes arginine into ornithine and urea and ultimately contributes to arginine deficiency. A reduced arginine concentration impairs T-cell and natural killer (NK) cells’ activity and proliferation, resulting in immunosuppression (68). Under arginine-deficient conditions, T cells will persistently downregulate CD3ζ, which can disturb TCR expression, reduce T cell proliferation, and decrease cytokine (e.g., IFN-γ, IL-5, IL-10) secretion. This ultimately contributes to a decrease activity of T cells (69). Arginine deficiency interferes with cell-cycle regulation, as T cells fail to upregulate key regulatory factors cyclin D3 and cdk4, leading to G0–G1 arrest, reduced Rb phosphorylation, and decreased E2F1 expression, which obstructs the proliferation of T cells (69). Moreover, arginine deficiency allows activated CD4^+^ T cells to acquire immunosuppressive properties like regulatory T cells (Tregs) via the ATF4-SLC7A11-GSH axis (70). These regulatory processes promote immunosuppression and tumor immune evasion.

#### 6.2.2 The IDO1-kynurenine-AhR axis

Chronic stress also activates the hypothalamic–pituitary–adrenal (HPA) axis, increasing the secretion of pro-inflammatory cytokines such as IFN-γ, which upregulates indoleamine 2,3-dioxygenase 1 (IDO1) expression (71). Elevated IDO1 expression level can induce immunosuppression, which may cause poor prognosis in squamous cell carcinoma patients (72). IDO1 is a key enzyme in the kynurenine pathway of tryptophan metabolism (71). Its metabolite kynurenine activates the aryl hydrocarbon receptor (AhR), which upregulates IL-10 expression and the population of Foxp3^+^ Tregs, thereby promoting immunosuppressive effects (73). IL-10 itself is an important immunosuppressive cytokine, playing a key role in alleviating inflammatory responses and promoting immune tolerance (74). IL-10 can inhibit inflammatory cytokines (e.g., IL-6, TNF-α), fostering an immunosuppressive microenvironment (74). IL-10 can also create a favorable microenvironment for Treg proliferation by inhibiting the expression of pro-inflammatory cytokines such as IL-6 and TNF-α (73). Furthermore, AhR itself can promote Foxp3 expression while suppressing CD8^+^ T-cell effector function (73, 75). These regulatory processes can reinforce immunosuppression.

### 6.3 Chronic stress regulates lipid metabolism, suppressing immunity and promoting tumor growth

#### 6.3.1 Fatty acid oxidation as a survival switch

Chronic stress–activated β2-AR signaling can reduce glycolysis while increasing oxidative phosphorylation and fatty acid oxidation (FAO) (76). It also increases the expression of the fatty acid transporter CPT1A (76). CPT1A-mediated fatty acid oxidation (FAO) in tumor cells acts as an “anti-scavenging switch. Inhibition or knockout of CPT1A gene significantly increased the sensitivity of tumor cells to CTL (cytotoxic T-cell) killing in multiple cancer models; in contrast, a sufficiently FAO process allowed tumor cells to maintain survival signals under “cytolytic stress” such as perforin/granzyme release by immune cells (77). In the tumor microenvironment, cytokines like IFN-γ induce tumor cells to upregulate CPT1A and FAO via AMPK, constituting a tumor-intrinsic response of “immune pressure → FAO adaptation → anti-scavenging” (77). FAO can mediate the immunosuppressive function of MDSC, thus counteracting the scavenging effect of immune cells (76). In addition, β2-AR signaling increases autophagy and activates the arachidonic acid cycle, both of which can increase PGE2 release, thereby suppressing the immune response (76).

#### 6.3.2 CD36-mediated lipid accumulation and Treg function

During early stages of stress, HPA axis– and sympathetic nervous system (SNS)-mediated upregulation of glucocorticoids (GCs) and catecholamines (CAs) increases proinflammatory cytokines (TNF-α, IL-6) and reduces anti-inflammatory cytokines, which can cause an inflammatory response (78). Studies have shown that inflammatory responses (mimicked by TNF-α and IL-6) will significantly increase hepatic CD36 protein levels both *in vitro* (HepG2 cells) and *in vivo* (mouse models) (79). An upregulation of CD36 can induce fatty acid oxidation and N-glycosylation of TβRII/IL-2Rα to promote the Treg response. And this will enhance the inflammatory response to create a positive feedback regulation (80). In cancer-associated fibroblasts (CAFs), CD36 regulates lipid uptake and extracellular matrix (ECM) production, which promotes tumor cell proliferation (81). CD36 upregulation also sustains FAO in MDSCs, preserving immunosuppressive function (82). In addition, CD36 promotes tumor cell adhesion to the extracellular matrix (ECM) and induces epithelial-mesenchymal transition (EMT) (82). CD36 can bind to TSP-1 and TSP-2, which can both inhibit tumor angiogenesis and promote tumor migration and invasion (82). CD36 can promote tumor angiogenesis through vascular mimicry (VM) (81). Inflammatory responses induced by chronic stress also promote the accumulation of lipid droplets, which closely associated with M2-type polarization in macrophages (83, 84). Lipid droplet formation and fatty acid oxidation provide energy support for M2-type macrophages, and M2 macrophages can lead to immunosuppression (83).

### 6.4 Chronic stress impairs mitochondrial metabolism, inducing oxidative stress and tumor progression

Chronic stress and persistent antigen stimulation can damage T-cell mitochondrial function, leading to excessive mitochondrial ROS accumulation and T-cell exhaustion (85). ROS can inhibit T-cell activity, impair TCR–MHC interactions, and promote immune evasion (86). ROS also induce Treg apoptosis, releasing immunosuppressive adenosine, further weakening immune function (86). Additionally, ROS can inhibit CD8^+^ T-cell proliferation, induce antigen-specific tolerance, and enhance PD-L1 expression via NF-κB activation to promote immune evasion (86, 87). Excessive ROS also impair dendritic cell (DC) function, reducing antigen presentation efficiency (87, 88). ROS can also stimulate production of immunosuppressive cytokines (IL-6, IL-10, TGF-β), thereby cause immunosuppression to promote tumor progression (88).

### 6.5 Chronic stress systemically weakens antitumor immune cells and activates immunosuppressive cells

Chronic stress can have a negative impact on the systemic immune system. These negative effects may ultimately allow cancer cells to evade immune responses, thereby promoting the occurrence and metastasis of cancer.

#### 6.5.1 Upregulation of immune checkpoints drives T cell exhaustion

Chronic stress exerts a multifaceted inhibitory effect on T cells. First, acting directly through β1-adrenergic receptors (β1-ARs), catecholamines suppress the secretion of IFN-γ and granzymes in CD8+ T cells, which are crucial for cytotoxic function and proliferation (27). Besides, chronic stress facilitates immune evasion by remodeling immune checkpoints. It activates the MAPK pathway via β-adrenergic signaling, simultaneously suppressing MHC-I expression while upregulating PD-L1 (89). Mechanistically, PD-L1 binds to PD-1 to recruit the tyrosine phosphatase SHP-2 via the ITSM domain, which inhibits key TCR signaling molecules, resulting in restricted metabolism, reduced cytokine production (e.g., IL-2, IFN-γ), and ultimately, T cell dysfunction (90, 91).

Beyond the PD-1 axis, chronic stress also engages other inhibitory pathways. It upregulates TIM-3 and its ligand Galectin-9, triggering autophagy-mediated apoptosis in CD4+ T cells and skewing the Th1/Th2 balance (92). Furthermore, stress-induced systemic inflammation (elevated IL-6, TNF-α) promotes LAG-3 expression. Specifically, IL-6 activates the STAT3 pathway to upregulate LAG-3 on CD8+ T cells (93, 94). The persistent co-expression of PD-1 and LAG-3 synergistically drives metabolic dysregulation and irreversible loss of effector function (95).

#### 6.5.2 Expansion of regulatory T cells via GR and TGF-βsignaling

Hormones related to chronic stress, such as cortisol, regulate immune cells by binding to intracellular glucocorticoid receptors (GR) (96). Studies show that GR plays a crucial role in regulatory T cells (Tregs). In mouse models lacking GR, Tregs are unable to effectively control experimental inflammatory bowel disease, which suggests that the GR signaling pathway is important for maintaining Foxp3 expression and Treg function (96). Furthermore, chronic stress involves the secretion of IL-35, an immunosuppressive cytokine specifically secreted by Foxp3+ Tregs and composed of two subunits, Ebi3 and IL-12α (p35) (97). IL-35 plays an important role in regulating immune responses, studies have shown that Tregs from mice with a lack of IL-35 (due to impaired Treg function from chronic stress) have significantly reduced *in vitro* and *in vivo* immunosuppressive capacity (97).

Additionally, chronic stress activates the TGF-β1/Smad2/3/Foxp3 signaling axis, causing a disruption in the dynamic balance of immune cells (98). Under stress conditions, DNA released from damaged tissues can be recognized by TLR9, activating the p38/MAPK pathway, thereby inducing the expression of pro-inflammatory factors and transforming growth factor-beta1 (TGF-β1) (98, 99). As a key immunoregulatory factor, TGF-β1 further activates the Smad2/3 signaling molecules, promoting the expression of the transcription factor Foxp3 in regulatory T cells (Tregs), which is a key marker for Treg function and differentiation (100). The activation of this signaling axis not only promotes the proliferation of Tregs but also induces the apoptosis of effector lymphocytes by upregulating pro-apoptotic signals, thereby weakening the body’s immune response capacity and leading to a state of immunosuppression (98).

In summary, chronic stress can cause immunosuppression by inhibiting the immune function of T cells, upregulating the function of regulatory T cells and increasing the secretion of the immunosuppressive cytokine IL-35. These ultimately lead to an immunodeficiency microenvironment to facilitate tumor progression.

#### 6.5.3 Glucocorticoid-mediated impairment of dendritic cell antigen presentation

Stress-related hormones from chronic stress can also affect the development and function of dendritic cells (DCs) (55). Studies show that under chronic stress, the sustained activation of the hypothalamic-pituitary-adrenal (HPA) axis leads to elevated levels of glucocorticoids (such as cortisol), which can induce the expression of the Tsc22d3 gene (also known as GILZ, glucocorticoid-induced leucine zipper) (101). As a downstream effector molecule of glucocorticoid signaling, Tsc22d3 can significantly inhibit the maturation of DCs, reduce the expression of co-stimulatory molecules on their surface (such as CD80, CD86, MHC II), and weaken their ability to present antigens to T cells, thereby inhibiting T cell activation and proliferation capacity (55, 101). This ultimately leads to immunosuppression and impaired antigen-specific immune responses, particularly affecting the immunogenicity and therapeutic efficacy of cancer vaccines (101). Related studies have further confirmed that high expression of Tsc22d3 in DCs can significantly inhibit their secretion of pro-inflammatory cytokines (such as IL-12), thereby creating an immunosuppressive microenvironment and weakening anti-tumor immunity (102).

#### 6.5.4 Modulation of macrophages and neutrophils to foster a pro-metastatic niche

The upregulation of the cAMP/PKA pathway caused by chronic stress can also activate the transcription factor CREB, which upregulates M2-type macrophage markers, such as Arginase-1 (Arg1) and mannose receptor (MRC1/CD206), promoting macrophage polarization towards the M2 phenotype (103). CCL18 is a chemokine secreted by M2-type TAMs and dendritic cells, which can induce the differentiation and recruitment of Tregs, thereby creating an immunosuppressive environment in the tumor microenvironment and promoting tumor progression (104).

Stress hormone like corticosterone can stimulate microglia to release HMGB1 (105). HMGB1 can interact with Toll-like receptor 4 (TLR4) to induce neutrophils to form NETs. After neutrophils are stimulated to form NETs, the amount of HMGB1 released significantly increases (106, 107). For NETs itself, it can capture circulating tumor cells through β1-integrin-mediated interactions and promote metastasis (108); Furthermore, HMGB1 released from NETs can activate TLR9 inside tumor cells, promotes the proliferation and migration of tumor cells through NF-κB-mediated IL-6 upregulation and the activation of the MAPK signaling pathway (106). Studies have found that under hypoxic stress conditions, HMGB1 translocates from the tumor cell nucleus to the cytoplasm and binds to TLR9, activating the p38/MAPK pathway, promoting PGC-1α phosphorylation, and enhancing mitochondrial biogenesis, thereby promoting the growth of liver cancer (109).

## 7 Influence of chronic stress on anticancer treatments from a immune perspective

Conventional cancer treatment strategies include surgical resection, chemotherapy and radiotherapy, which have been employed in clinical practice for decades, while these approaches are often associated with significant adverse effects (110, 111). In recent years, with the development of technology, novel immunotherapies and targeted therapies have appeared. When used as an adjunct to these conventional treatments, these modern approaches have substantially mitigated adverse reactions (112, 113). Despite their widespread clinical application, clinical evidence indicates that chronic stress negatively impacts the efficacy of several of these treatment modalities, including chemotherapy, radiotherapy, immunotherapy, and targeted therapy.

### 7.1 The influence mechanism of chronic stress on chemotherapy efficacy

Current chemotherapeutic agents are primarily classified as alkylating agents, antimetabolites, topoisomerase inhibitors and mitotic spindle inhibitors to exert their cytotoxic effects by damaging DNA or disrupting the cell cycle to treat tumors (114). These agents are widely employed in clinical tumor treatment, but their therapeutic efficacy is often compromised in patients exhibiting multidrug resistance (MDR), a major obstacle in cancer therapy (115). Emerging evidence suggests that chronic stress plays a pivotal role in the development of MDR.

Chronic stress can activate β-ARs on tumor cell surfaces, upregulating the expression of lactate dehydrogenase A (LDHA), which catalyzes the conversion of pyruvate to lactate and contributes to acidification of the TME (116). Subsequently, this acidic environment not only impairs the function of immune cells but also compromises the efficacy of various drugs (117). For instance, alkaline drugs such as anthracyclines are particularly sensitive to extracellular pH, losing the lipophilicity required to traverse the cell membrane because of protonation in acidic environment, so that they cannot finally enter into the tumor cells (118). Additionally, breast cancer resistance protein (BCRP), as an ATP-dependent efflux transporter, mediates the transport of various hydrophobic compounds, including a wide range of anticancer drugs, and the BCRP is overexpressed under acidic conditions within the TME (119, 120). Collectively, these adaptations reduce intracellular drug accumulation by both limiting cellular uptake and enhancing drug efflux, thereby contributing to the development of MDR (115).

### 7.2 The influence mechanism of chronic stress on radiotherapy efficacy

The primary mechanism of radiotherapy involves the delivery of high-energy ionizing radiation to tumor cells (121). Radiotherapy induces DNA double-strand breaks (DSBs) to result in direct genomic damage (122). It can also generate uncontrolled lethal reactive oxygen species (ROS) burst to indirectly contribute to cytotoxicity by compromising genomic integrity and finally suppressing tumor cell proliferation (123). However, both of these cytotoxic pathways can be attenuated by chronic stress through the action of NE. As a catecholamine, it upregulates tripartite motif-containing protein 2 (TRIM2) expression in tumor cells, leading to the ubiquitination-mediated degradation of I-κBα and subsequent activation of the NF-κB signaling pathway (124). Consequently, elevated NF-κB levels lead to the overexpression of cyclooxygenase-2 (COX-2) and matrix metalloproteinase-2 (MMP-2), while downregulating caspase-3 production to increase cell viability and inhibits apoptosis, thereby counteracting the effects of radiotherapy (125).

### 7.3 The influence mechanism of chronic stress on emerging anticancer therapies efficacy

The advent of novel anticancer therapies, such as immunotherapy and targeted treatments, which have markedly improved the efficacy of cancer treatment, when integrated with conventional approaches. Under normal conditions, tumor cells are eliminated by the innate and adaptive immune systems during the early stages of tumorigenesis, with CD8+ T cells playing a primary role in this process (126). However, tumor cells can evade immune surveillance and progress into malignant disease (127). This primary mechanism of tumor immune evasion is the upregulation of immune checkpoints like programmed cell death ligand 1 (PD-L1) and cytotoxic T-lymphocyte-associated protein 4 (CTLA-4), as well as major histocompatibility complex (MHC) class molecules, which effectively hide tumor cells from the immune system’s effector T cells (128). Consequently, current major therapeutic strategies focus on blocking these pathways, including the use of immune checkpoint inhibitors (ICIs), cancer vaccines and other methods to enhance the immune system (129). However, stress hormones present a significant challenge to these therapies. Acting through β1-ARs, stress hormones can phosphorylate eIF2α and activate p38 MAPK, which leads to elevated PD-L1 levels, while the STAT1/IRF1 axis is also engaged to suppress the processing and presentation of antigens via MHC class I molecules simultaneously (89). This upregulation of PD-L1 directly undermines the effectiveness of ICIs, while the lack of MHC-I negatively impacts the efficacy of cancer vaccines. Furthermore, the potent immunosuppressive properties of GCs exacerbate these challenges by broadly limiting immune activation (130, 131).

In the context of targeted therapies, these agents are designed to interact with specific molecular targets that are critically involved in oncotherapy. Compared to conventional treatments, the high specificity of these drugs significantly reduces common systemic toxicities, while this safety advantage allows for long-term treatment, mitigating the intensity of cumulative adverse effects (132). Among these targets, the epidermal growth factor receptor (EGFR) is a prominent therapeutic focus, and the efficacy of EGFR tyrosine kinase inhibitors (TKIs) can be compromised by NE (133). With the combination between β2-Ars and NE, the levels of the cAMP response element-binding protein (CREB) are upregulated and then increasing expression of the transcription factor Cx32 which modulates key proteins associated with EGFR-TKI resistance, such as MET and IGF-1R (133, 134). Consequently, stress hormones can promote resistance to EGFR-TKIs like afatinib via β2-ARs. Importantly, this pathway presents a potential opportunity for intervention, as this resistance can be counteracted through the use of β-blockers.

## 8 Therapy targeting chronic stress

Chronic stress mediated by stress hormone receptor signaling pathways is a significant driver of immunosuppression that ultimately results in immune evasion and enhanced metastatic potential. Consequently, targeting key neuroendocrine pathways has become a crucial strategy to elevate cancer prognosis. A growing number of evidence has demonstrated that β-AR antagonists can effectively block the pro-tumorigenic responses mediated by chronic stress and exhibit broad-spectrum anti-tumor activity across various tumor models (135, 136). Among them, the classical agent propranolol has been shown to reverse tumor treatment resistance (137). Among the multiple cancer metastasis models, propranolol blocks the β2-ARs on monocytes facilitates their functional maturation with upregulated expression of MHC class II to counteract the immunosuppressive effect driven by chronic stress (138). These modified monocytes exhibit enhanced tumor-antigen presentation efficiency, which drives CD4+ T cells into a cytotoxic Th1 phenotype with direct tumoricidal capabilities (138). Similarly, the active component of the traditional Chinese medicine Radix bupleuri, saikosaponin D, also functions to reverse drug resistance modulating glucose metabolism and drug efflux through the inhibition of β2-AR signaling (139). Whether employed as a primary or adjunctive therapy, β-AR blockers have shown remarkable clinical potential (140). Meanwhile, nuclear receptor antagonists targeting glucocorticoid signaling have also demonstrated therapeutic activity in various malignancies (141). In stance, highly selective agents, such as ORIC-101, have exhibited synergistic effects when combined with conventional chemotherapeutics (e.g., paclitaxel), further highlighting their translational promise (141, 142).

Beyond direct antagonism of stress hormone receptors, a complementary and critical strategy is to intervene at the source by targeting chronic stress itself (143). Existing researches suggest that antidepressant therapies may exert cross-indication anti-tumor potential in cancer treatment, though their underlying mechanisms and clinical value require further investigation (144, 145). For example, the selective serotonin reuptake inhibitors (SSRIs), as a common class of conventional antidepressants, have been shown to improve the anti-tumor immune response by enhancing cytotoxic T-cell activity through the inhibition of the serotonin transporter (SERT) (146). The inhibition of the SERT disturbs the homeostatic reuptake of 5-HT, resulting the extracellular accumulation of this monoamine within the interstitial spaces of the central nucleus in the amygdala to inhibit the release of CRH (147). It directly suppresses the over-reaction of HPA axis under the effect of chronic stress. However, the long-term use of SSRIs may present severe adverse effects, including serotonin syndrome and hyponatremia, which pose significant concerns regarding chronic administration safety (148). Beyond these classical antidepressants, several natural plant-derived bioactive compounds have been considered as potential alternatives due to their lower toxicity and antidepressant effects (149, 150). Quercetin can downregulate the voltage-gated calcium channel subunit α2δ-1, which in turn disrupts its interaction with the N-methyl-D-aspartate receptor (NMDAR) to finally achieve the resistant against depression (151, 152). Because of the disconjunction between the α2δ-1 and NMDAR, the synaptic localization of NMDARs and NMDAR-mediated excitatory Ca²^+^ influx are reduced, leading to decrease the CRH release by inhibiting excitability of CRH neuron (151). Similarly, the St. John’s wort (SJW) extract Ze 117 has been widely recognized as a natural alternative for synthetic antidepressants, which has demonstrated well-defined antidepressant efficacy (153). As a potent inhibitor of neurotransmitter, the SJW enhance the synaptic availability of 5-HT, dopamine, GABA and glutamate, which contributes to maintain the central neurotransmitter homeostasis under chronic stress, also partially reversing the excessive activation of the HPA axis caused by chronic stress the central level (154).

In addition, psychosocial and behavioral interventions provide a non-pharmacological novel strategy to counteract the pro-tumorigenic effects induced by chronic stress by restoring the homeostasis of the neuroendocrine–immune axis. The softer approaches such as music therapy, massage, aromatherapy and acupuncture have been shown in clinical trials to alleviate depressive and anxious symptoms while reducing dependence on antidepressant medication (155). Moreover, the mindfulness-based stress reduction (MBSR) has been demonstrated to significantly decrease psychological stress levels. Its mechanism is thought to involve inhibition of HPA axis activity and subsequent reduction in glucocorticoid levels, thus exhibiting clear translational potential for clinical application (156, 157).

## 9 Conclusion and perspectives

Chronic stress, driven by persistent HPA axis and SNS activation, promotes tumor progression by crippling anti-tumor immunity. Elevated stress hormones act through multiple pathways to foster an immunosuppressive tumor microenvironment, disrupt systemic immune surveillance, and induce oxidative damage. Together, these effects not only accelerate tumor growth but also compromise the efficacy of standard therapies. Therefore, targeting the neuroendocrine-immune crosstalk represents a promising frontier for improving cancer outcomes.

Translating these mechanistic insights into clinical practice requires rational intervention strategies. Pharmacologically, β-adrenergic receptor blockers and glucocorticoid receptor antagonists have shown potential in countering treatment resistance and improving outcomes. In contrast, antidepressants aim to mitigate the central drive of chronic stress. Furthermore, psychosocial and behavioral interventions provide a non-pharmacological approach to normalizing stress hormone levels, often with a superior safety profile. However, a unified strategy is needed to guide the selection and integration of these modalities. To this end, we propose a tiered screening and intervention model. This framework utilizes biomarkers such as salivary cortisol to stratify patients based on their stress burden, enabling the personalized application of the aforementioned strategies.

Although targeting chronic stress presents a promising therapeutic avenue, several challenges remain. Firstly, current mechanistic insights are derived predominantly from preclinical models, which cannot fully recapitulate the complexity of the human tumor microenvironment. Secondly, the universality of these mechanisms across diverse cancer types is still uncertain. Future research should prioritize integrating large-scale clinical and genomic data to identify conserved stress-responsive pathways, followed by validation in more human-relevant models. Additionally, the mechanistic basis of psychosocial interventions and the fundamental neurobiology of sustained stress axis activation are poorly understood. Addressing these gaps is essential to transition from symptom management to targeted intervention. Finally, the proposed tiered intervention model, while promising, requires rigorous clinical validation and standardized assessment criteria to establish its utility. Overcoming these hurdles will be crucial for integrating stress modulation as a credible dimension of comprehensive cancer care.
